# A Novel Protein Hydrolysate-Based Biostimulant Improves Tomato Performances under Drought Stress

**DOI:** 10.3390/plants10040783

**Published:** 2021-04-16

**Authors:** Silvana Francesca, Valerio Cirillo, Giampaolo Raimondi, Albino Maggio, Amalia Barone, Maria Manuela Rigano

**Affiliations:** Department of Agricultural Sciences, University of Naples Federico II, Portici, 80055 Naples, Italy; silvana.francesca@unina.it (S.F.); valerio.cirillo@unina.it (V.C.); giampaolo.raimondi@unina.it (G.R.); almaggio@unina.it (A.M.); ambarone@unina.it (A.B.)

**Keywords:** water shortage, yield, glycine betaine, proline, pollen viability, fruit set

## Abstract

Abiotic stresses adversely affect crop production causing yield reductions in important crops, including tomato (*Solanum lycopersicum* L.). Among the different abiotic stresses, drought is considered to be the most critical one, since limited water availability negatively impacts plant growth and development, especially in arid and semi-arid areas. The aim of this study was to understand how biostimulants may interact with critical physiological response mechanisms in tomato under limited water availability and to define strategies to improve tomato performances under drought stress. We investigated the physiological responses of the tomato genotype ‘E42’ grown in open fields under optimal conditions (100% irrigation) and limited water availability (50% irrigation) treated or not with a novel protein hydrolysate-based biostimulant (CycoFlow, Agriges, BN, Italy). Plants treated with the protein hydrolysate showed a better water status and pollen viability, which also resulted in higher yield under drought stress compared to untreated plants. The treatment with the biostimulant had also an effect on antioxidant contents and activity in leaves and fruits depending on the level of irrigation provided. Altogether, these results indicate that the application of protein hydrolysates on tomato improved plant performances under limited water availability and in different experimental fields.

## 1. Introduction

Tomato is one of the most important crops, with more than five millions hectares cultivated, worldwide [1]. Being a summer crop, transient or extended drought periods are common during its cultivation cycle, especially in the most sensitive periods of fruit set and enlargement [2]. These are the two phases in which even a transitory drought stress can lead to heavy yield losses [3]. Climate change will make these events more frequent in arid and semi-arid environments, with detrimental consequences for tomato productivity [4,5]. Generally, plants respond to drought with a series of physiological mechanisms including stomatal closure, repression of cell growth and photosynthesis, and activation of stress hormones and antioxidant mechanisms, which overall lead to a reduction in plant growth and productivity [6]. The forecasted lack of water and the consequent increase in competition for water resources between agriculture and other sectors require the exploration of alternative and sustainable crop management strategies that can save water for irrigation and, at the same time, still maintain satisfactory levels of crop production [7].

One of the most promising strategies that can be used to improve plant response to drought stress is the use of biostimulants. These are substances or micro-organisms whose application is beneficial for plant growth and productivity [8]. The application of biostimulants can also induce enhanced tolerance to different abiotic stresses [9,10]. Among biostimulants, protein hydrolysates seem to be promising, since they contain high amounts of molecules such as amino acids, small peptides and osmoactive compounds (proline, glycine betaine) which are beneficial for plant productivity under unfavorable environmental conditions [9]. Plant-based biostimulants are also effective in enhancing growth, yield, quality and bioactive compounds’ content in various crops. For example, the application of an extract from moringa (*Moringa oleifera* Lam.) increased yield and growth in tomato, basil, cabbage and pepper, as well as the quality of tomato, lettuce, radish, spinach, rocket and pepper [11]. These extracts also improved plant tolerance to different abiotic stresses such as drought [12], salinity [13], heat [14] and heavy metal contamination [15]. Ertani et al. [16] found that foliar application of alfalfa (*Medicago sativa* L.) and red grape (*Vitis vinifera* L.) extracts improved growth and yield in *Capsicum chinensis* L., and triggered the accumulation of secondary metabolites in leaves. On rocket, the use of two vegetal-based biostimulants enhanced productivity under both optimal and sub-optimal nitrogen fertilization rates [17].

Despite the high number of scientific papers in which protein hydrolysates and plant-based biostimulants have been proven to increase crop productivity and abiotic stress tolerance, the functional cause–effect relationship and physiological basis that determine the growth stimulant and/or protective action of these products is still unclear [2].

The aim of this study was to link the physiological responses and agronomic performances of tomato plants treated with a plant-derived protein hydrolysate. This biostimulant (CycoFlow, Agriges, Benevento, Italy) was previously found to be effective in heat stress protection on different tomato varieties grown in open field. In this study, we tested whether the application of this biostimulant could be beneficial for tomato productivity and fruit quality under two different water regimes (optimal and water limited) and in different environmental fields. The physiological bases of these responses are discussed.

## 2. Results

### 2.1. Biomass and Yield Components

**Figure 1:**
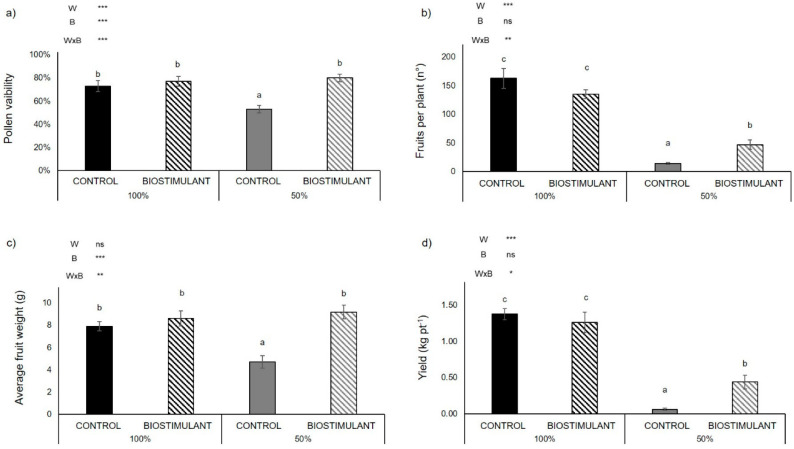
Pollen viability (**a**); number of fruits per plant (**b**); average fruit weight (**c**) and yield *per* plant (**d**) in the tomato genotype ‘E42’ grown in open field in Battipaglia under optimal (100% irrigation) and limited water availability (50% irrigation) and treated (biostimulant) or not (control) with the biostimulant. Values are mean ± SE. Asterisks indicate significant effect of limited water availability (W), biostimulant treatment (B) and their interaction (W × B) according to ANOVA (ns = not significant; * = *p* < 0.05; ** = *p* < 0.01; *** = *p* < 0.001). Different letters indicate significant differences based on Duncan’s test (*p* ≤ 0.05).

We investigated the performances of one tomato genotype (‘E42’) grown in the year 2019 in an open field located in Battipaglia in the Campania Region (Italy) under optimal conditions (100% irrigation) and limited water availability (50% irrigation), and treated or not with a protein hydrolysate-based biostimulant. Pollen viability decreased by 27% under water deficit in non-treated plants. On the contrary, plants treated with the protein hydrolysate and subjected to water deficit showed an increase of 51% in pollen viability compared to non-treated plants (Figure 1a). Water deficit significantly reduced the number of fruits per plant. The biostimulant treatment partially compensated the effect of water deficit (50% water regimen), as demonstrated by the 70% higher number of fruits per plant upon biostimulant treatment vs. non-treated plants (Figure 1b). The treatment with the biostimulant increased the average weight of a single fruit under reduced water regimen by 95% (Figure 1c). Under the 50% water regimen, biostimulant treatment increased the final yield six-fold (Figure 1d). Moreover, treatment with the protein hydrolysate increased the fruits’ water content under reduced water regimen (98% vs. 91%—Table S1). According to ANOVA, the combined effect of water stress and biostimulant did not induce significant differences in shoot fresh weight. Conversely, the single effect of the water stress induced a significant reduction in this parameter, while the biostimulant treatment had an opposite effect (Table S1).

In order to further confirm the effect of the protein hydrolysate on plant growth and on final yield and yield components, an additional experiment was carried out in the year 2020 in another experimental field located in Benevento (Campania Region, Italy) on plants grown in optimal conditions (Table 1). These analyses evidenced the positive effect of the protein hydrolysate on pollen viability, number of fruits and final yield (+112% in treated plant compared to non-treated ones). Moreover, considering the fresh biomass accumulation, the biostimulant treatment induced a higher shoot fresh weight in this experimental field (Table 1).

### 2.2. Physiological Traits

**Figure 2:**
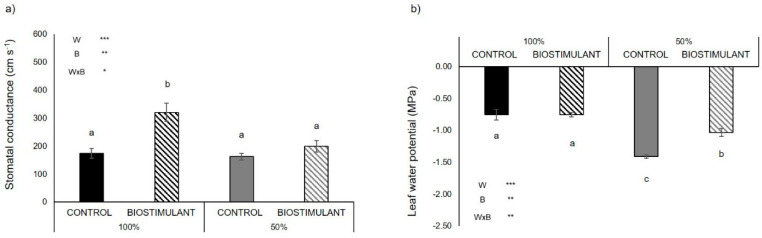
Stomatal conductance (**a**); and leaf water potential (MPa) (**b**); in the tomato genotype ‘E42’ grown in open field in Battipaglia under optimal (100% irrigation) and limited water availability (50% irrigation) and treated (biostimulant) or not (control) with the biostimulant. Values are mean ± SE. Asterisks indicate significant effect of limited water availability (W), biostimulant treatment (B) and their interaction (W × B) according to ANOVA (ns = not significant; * = *p* < 0.05; ** = *p* < 0.01; *** = *p* < 0.001). Different letters indicate significant differences based on Duncan’s test (*p* ≤ 0.05).

In the plants grown in the open field located in Battipaglia under optimal conditions (100% irrigation) and limited water availability (50% irrigation), treatment with the biostimulant had a significant effect on stomatal conductance, which, under full irrigation, increased by 84% after treatment (Figure 2a). The 50% water regimen significantly reduced the leaf water potential compared to plants under full irrigation; however, the treatment with the protein hydrolysate led to a 27% increase in the leaf water potential compared to non-treated plants under water deficit, confirming beneficial effects in terms of plant water status (Figure 2b).

### 2.3. Leaf Antioxidant Activity

**Figure 3:**
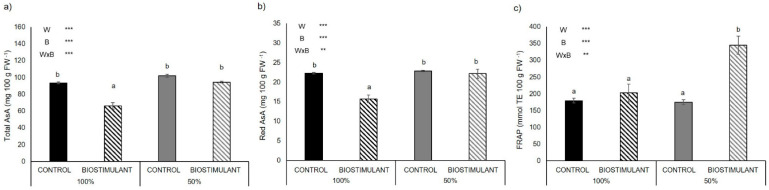
Total (**a**) and reduced (Red) (**b**) ascorbic acid (AsA) content and total antioxidant activity (FRAP) (**c**) in leaves of tomato genotype ‘E42’ grown in open field in Battipaglia under optimal (100% irrigation) and limited water availability (50% irrigation) and treated (biostimulant) or not (control) with the biostimulant. Values are mean ± SE. Asterisks indicate significant effect of limited water availability (W), biostimulant treatment (B) and their interaction (W × B) according to ANOVA (ns = not significant; * = *p* < 0.05; ** = *p* < 0.01; *** = *p* < 0.001). Different letters indicate significant differences based on Duncan’s test (*p* ≤ 0.05).

The content of total and reduced ascorbic acid (AsA) in the leaves of the plants grown in the open field located in Battipaglia was significantly reduced by biostimulant treatment only under 100% irrigation (−29% for both reduced and total AsA) (Figure 3a,b). Under limited water availability, the total antioxidant activity (FRAP) in the leaves increased by 98% after treatment with the biostimulant, while no change was reported under full irrigation (Figure 3c). Overall, the 50% water regimen increased carotenoid content. The interaction between water regimen and biostimulant treatment significantly reduced the content of chlorophylls a and b by 14% under full irrigation (Table S2).

### 2.4. Fruit Antioxidant Activity

**Figure 4:**
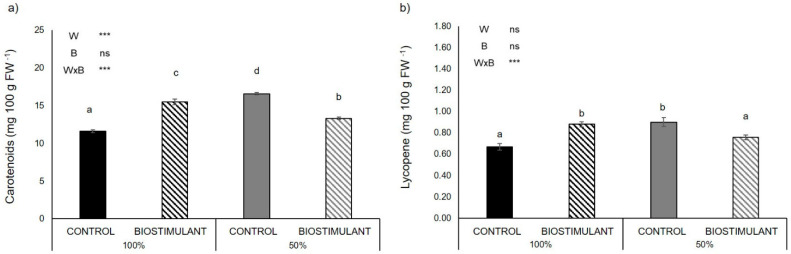
Content of (**a**) carotenoids and (**b**) lycopene in fruit of the tomato genotype ‘E42’ grown in open field in Battipaglia under optimal (100% irrigation) and limited water availability (50% irrigation) and treated (biostimulant) or not (control) with the biostimulant. Values are mean ± SE. Asterisks indicate significant effect of limited water availability (W), biostimulant treatment (B) and their interaction (W × B) according to ANOVA (ns = not significant; * = *p* < 0.05; ** = *p* < 0.01; *** = *p* < 0.001). Different letters indicate significant differences based on Duncan’s test (*p* ≤ 0.05).

In fruits of the plants grown in the open field located in Battipaglia carotenoid and lycopene contents were significantly affected by the interaction between biostimulant treatments and water regimen (Figure 4). In particular, under the 100% water regimen, both carotenoid and lycopene contents in fruits were significantly increased by biostimulant treatment (+33% and +31%, respectively). Under the 50% water regimen, this trend was inverted, with 20% and 15% lower carotenoids and lycopene accumulation in the fruits of treated plants compared to non-treated ones (Figure 4). Comparing non-treated plants, water stress caused an accumulation of carotenoids and lycopene (+43% and +34%, respectively) (Figure 4). The treatment with the biostimulant reduced the content of total ascorbic acid both under full irrigated (−12%) and water limited (−8%) conditions (Table S2). The content of reduced ascorbic acid was significantly affected by the interaction between water regimen and biostimulant treatments, with 10% reduction in plants treated with the biostimulant under full irrigation (Table S2). Finally, only the effect of the reduced water regimen induced significant changes in β-carotene accumulation and total antioxidant activity (Table S2).

### 2.5. Heat Map Analysis

**Figure 5:**
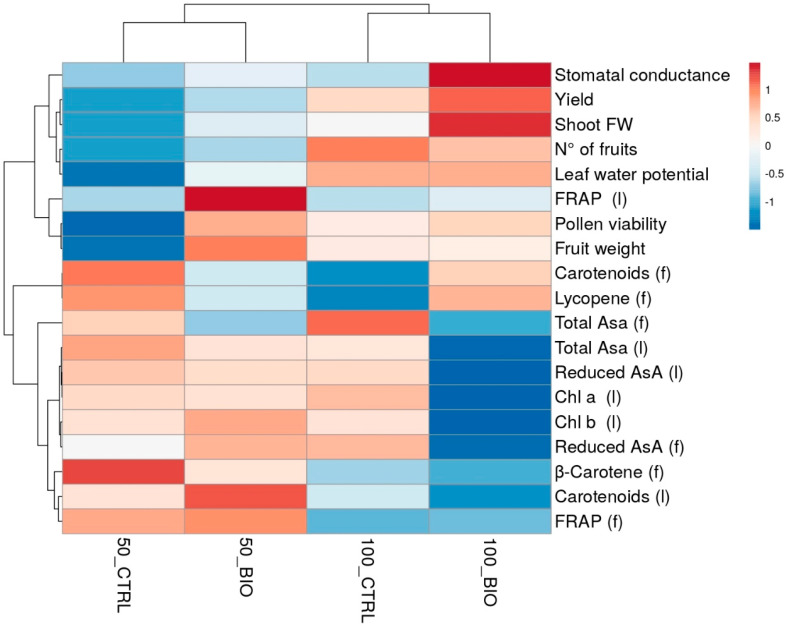
Heat map analysis summarizing plant responses to biostimulant application and water deficit in plants grown in Battipaglia under optimal (100) and limited water availability (50) and treated (BIO) or not (CTRL) with the protein hydrolysate. The letters in brackets indicate measurements taken from leaves (l) and fruits (f). The Figure was generated using the http://biit.cs.ut.ee/clustvis (accessed on 1 April 2021) program package with Euclidean distance as the similarity measure and hierarchical clustering with complete linkage.

The aggregated data heat-map analysis (Figure 5), summarizing plant responses to biostimulant application and water deficit in plants grown in Battipaglia, identified a first cluster corresponding to the different water regimen applied. Two separate sub-clusters could be defined under each single water treatment, which basically depended on the treatment with the biostimulant.

## 3. Discussion

### 3.1. A Protein Hydrolysate-Based-Biostimulant Protects Pollen Viability from Drought-Induced Desiccation

In this study, we investigated the performances of one tomato genotype (‘E42’) grown in open fields located in Battipaglia and in Benevento in the Campania Region (Italy), treated with a protein hydrolysate rich in glutamic acid, glycine betaine and micronutrients as boron, manganese and zinc. Biostimulants have demonstrated beneficial effects in promoting growth and alleviating the effects of abiotic stresses in horticultural crops [18]. Herein, we demonstrated, for two years and in different experimental fields, the positive effect of the protein hydrolysate-based biostimulant on plant growth, final yield and yield components (Figure 1, Table 1), in agreement with previous results [14]. Additionally, we tested the effect of the protein hydrolysate in plants grown under optimal conditions (100% irrigation) and limited water availability (50% irrigation). Both the water regimen and the biostimulant treatment had an effect on tomato plants, as shown by the heat map of Figure 5. Plant growth and yield were reduced in water-stressed plants compared to well-irrigated ones, but after treatment with the protein hydrolysate, both well-watered and water-stressed plants showed better performance in the field. Altogether, plants treated with the protein hydrolysate showed higher shoot biomass under both well-watered and water shortage conditions and in both experimental fields (Table 1 and Table S1), pointing out at the double effect of this biostimulant as a growth-promoting and stress-protective effector on plants. More interestingly, we found that treated plants showed higher pollen viability compared to non-treated ones under drought in 2019 and also under optimal conditions in 2020 (Figure 1a, Table 1). Pollen viability has been recently used to identify heat-tolerant tomato genotypes, indicating pollen thermo-tolerance as an important parameter to consider for future breeding programs. Shen et al. [19] indicated that the β-alanine plays a role in pollen germination under high temperatures. It is possible that the high concentration of this molecule, which is the third most representative free amino acid contained in the biostimulant (Table S3), was responsible for the higher pollen viability found in the biostimulant-treated plants. Other than heat stress, pollen viability is highly sensitive to drought and salinity [20,21,22,23]. Under several abiotic stresses, indeed, the concentration of reactive oxygen species (ROS) increases and leads to oxidative stress, which irreversibly damages pollen, thus reducing its viability and development [24,25,26,27]. The reduction in pollen viability and germination is generally correlated with yield losses, since it is tightly correlated with plant productivity and contributes to determining the level of fruit/seed set [28,29,30].

### 3.2. Plant Yield Improves upon Biostimulant Treatment

In this study, we found a higher number of fruits in treated plants compared to non-treated ones under drought in 2019, and also under optimal conditions in 2020 (Figure 1b, Table 1). This can be the direct consequence of the higher pollen viability induced by biostimulant treatment, and thus one of the reasons for the higher yield of treated plants compared to non-treated ones (Figure 1a,d, Table 1). Moreover, the biostimulant treatment increased the average weight of single tomato fruits compared to untreated plants under drought (Figure 1c). Therefore, other than the enhancement of fruit set, the protein hydrolysate reduced the drought-induced fruit shrinkage. This can be the result of the better water status of the treated plants under drought compared to the non-treated ones, as indicated by their higher leaf water potential (Figure 2b) and the different fruit water content, which was significantly higher in the former compared to the latter (98% vs. 91%—Table S1). Indeed, leaf water potential is an indicator of plant water status [31,32], thus underlying that plants treated with the biostimulant were less sensitive to water deprivation compared to non-treated ones. It is possible that the presence of protective metabolites such as glycine betaine and proline in the protein hydrolysate may have enhanced the tolerance of tomato plants to water deficit. Indeed, it has been previously demonstrated that both glycine betaine and proline applied exogenously significantly increased drought tolerance of tomato plants grown under hyper-osmotic conditions, thanks to different mechanisms such as osmotic adjustment, membrane and proteins stabilization, and antioxidant activity [33,34]. Moreover, it is known that the amino acid proline also favors the translocation of nutrients towards developing flowers (sink) [35].

### 3.3. Plant Antioxidant Activity of Leaves Is Enhanced by Biostimulant Treatment

The biostimulant had a positive effect on the total antioxidant activity (FRAP) in leaves of plant grown under limited water availability (Figure 3c). The higher total antioxidant activity found in treated plants under water stress was not due to an increase in ascorbic acid, since the content of this antioxidant was significantly reduced by biostimulant application (Figure 3a,b). Possibly, treated plants had a lower need for ascorbic acid thanks to the exogenous application of molecules contained in the biostimulant formulation, such as glutamic acid, phenylalanine, glycine and proline, which can perform antioxidant activity when accumulated in plant tissues [36,37]. These amino acids are found in high concentrations in the formulation of the protein hydrolysate used in this study (Table S3) [14]. In this experiment, it is probable that these metabolites worked both as compatible solutes, thus improving plant water status under drought and promoting cell enlargement, as well as antioxidant production, preventing reactive oxygen species (ROS) damage to pollen viability, with a beneficial effect on fruit set. Indeed, enzymatic and non-enzymatic antioxidant system can reduce the oxidative stress of the membranes, thus increasing the pollen integrity, viability and pollen tube development [38,39]. These results are consistent with a previous work, which demonstrated that treatment with this biostimulant induced the activation of the antioxidant defense system [14]. The improved water status and the protection of cellular membranes under drought could be the reason for the higher yield reported in treated plants, which was mediated by the higher drought tolerance of these plants during the sensitive stages of fruit set and enlargement. Moreover, the free amino acids present in the biostimulant may have acted as signaling molecules and may have promoted endogenous phytohormonal biosynthesis, thus stimulating plant growth and productivity [40].

### 3.4. Plant Antioxidant Activity of Fruits Is Enhanced by Biostimulant Treatment

Regarding fruit quality, the content of carotenoids and lycopene was higher in the fruit of treated plants compared to the non-treated ones under well-watered conditions (Figure 4). These results are in agreement with the results previously obtained by Rouphael et al. [40], who showed that foliar applications of a protein hydrolysate derived from legumes had a similar effect on the lycopene content in tomatoes. Fruit vegetables, particularly tomatoes, are considered good sources of lipophilic and hydrophilic antioxidant molecules such as lycopene and ascorbic acid [41]. The beneficial effects of plant-based biostimulant on the accumulation of phytochemical compounds (i.e., lycopene) could be associated with the activation of specific molecular and physiological mechanisms related to nitrogen metabolism [16,42]. In conditions of limited water availability, plants react with an increase in the content of carotenoids. According to Riggi et al. [43], tomato plants subjected to mild water stress increase the content of lycopene and β-carotene compared to well-irrigated plants. It is conceivable that this protein hydrolysate, which contains two main osmolytes involved in osmotic stress, acts as a mild stressor to the plant. This could be the reason for the increase in lycopene content in the fruit of treated plants compared to non-treated ones under well-watered conditions, which equals the concentration of this antioxidant in the fruits grown under limited water availability (Figure 4). The higher lycopene content of treated fruits is valuable in view of the necessity to increase the nutraceutical properties of vegetables products, which are important components to supporting human health [44]. On the contrary, under water limited conditions, the biostimulant treatment reduced carotenoids and lycopene concentration when compared to untreated plants (Figure 4). This could have been induced by the higher water content of the fruit from biostimulant treated plants, which diluted the concentration of these antioxidants, compared to the ones from untreated plants. These contrasting results underline the fact that it is important to evaluate the specific effects of the biostimulant products depending on the condition of its application in order to maximize the desired effect of its employment.

## 4. Materials and Methods

### 4.1. Plant Growth, Experimental Design, and Treatments

One experiment was carried out at the agronomy farm of the University of Naples “Torre Lama” located in Battipaglia, Salerno, Italy (latitude 40°31′ N; longitude 14°58′ E) on a clay-loam soil. Four weeks after seeding, at the third true leaf fully expanded, tomato plants (genotype ‘E42’, available at the University of Naples, Department of Agricultural Sciences [45]) were transplanted in an open field on 19 June 2019. Rainfall throughout the growing period was 10 m^3^ ha^−1^ and mean daily air temperature was between 17 and 27 °C (Figure S1). The experimental design consisted of four treatments: non-treated plants, biostimulant-treated plants and two irrigation levels (100% replenishment of crop water requirements (CWR) estimated using a Class A evaporation pan vs. 50% CWR). Plants were arranged in a completely randomized block design with three replicates per treatment and 20 plants per biological replication. The experimental field was irrigated every 10 days, using a drip irrigation system with 5 L h^−1^ (one emitter per plant). Water deficit was induced at 22 Days After Transplant (DAT) and continued until the end of the experiment. The biostimulant was applied by fertigation at a concentration of 3 g/l of water (400 mL *per* plant) at transplanting and, thereafter, every 15 days until the end of the cultivation cycle for a total of four applications. The biostimulant tested was CycoFlow, a protein hydrolysate produced by Agriges (Benevento, Italy) by mixing sugar cane molasses with yeast extract obtained by autolysis of previously grown *Saccharomyces cerevisiae* yeasts. Its composition was previously reported [14]. Additional details on the composition of the biostimulant and the aminogram are reported in Table S3. According to the classification of the different biostimulants provided from du Jardin et al. 2015 [18], which define the protein hydrolysates category as “amino-acids and peptides mixtures obtained by chemical and enzymatic protein hydrolysis from agroindustrial by-products, from both plant sources (crop residues) and animal wastes (e.g., collagen, epithelial tissues)” we can define the product used in this study as a protein-hydrolysate. Harvesting started on 12 August 2019, at 54 DAT, on six plants per biological replicate per treatment.

In order to further confirm the effect of the protein hydrolysate-based biostimulant on plant growth and on final yield, a second experiment was carried out in the year 2020 in another experimental field (Table 1). The experimental field was located in an agronomy farm in Apollosa, (Benevento, Campania, Italy, latitude 41°5′42″36 N; longitude 14°42′22″32 E) characterized by a clay-loam soil. Four weeks following seeding, after the third true leaf was fully expanded, tomato plants (genotype ‘E42’) were transplanted into an open field in May 2020. Rainfall throughout the growing period was 344 m^3^ ha^−1^ and mean daily air temperature was between 14 and 29 °C (Figure S1). Tomato plants were grown following the standard agronomical practices of the area. The experimental design consisted of a completely randomized design with three replicates per treatment and 10 plants per biological replication. There were two different groups: one control, which did not receive any biostimulant, and one which was treated with the biostimulant. The same methods and quantities used in the first experiment were also maintained in the second year of the experiment.

### 4.2. Biometric, Yield and Physiology Measurements

Shoot biomass was calculated as the sum of above-ground vegetative plant parts (leaves + stems) in both experimental years. The number of fruits, the average single fruit weight and their total biomass were recorded in each experiment. During the cultivation cycle, the confirmation of plant stress was obtained measuring stomatal conductance and leaf water potential after 45 DAT (25 day after stress induction). Following procedures reported in other works [46,47,48,49], stomatal conductance was measured with a steady state porometer (AP-4, Delta-T Devices, Cambridge, UK) on a young and healthy, fully expanded apical leaf of the third branch per plant. The value derived from the average of three measurements in different positions of the leaf abaxial side of the selected leaf. On the same leaf used for stomatal conductance measurements, the total leaf water potential (Ψt) was measured with a Scholander’s pressure chamber (PMS Instrument Company, Albany, NY, USA), following procedures reported in other works [46,47,48,49].

### 4.3. Pollen Viability

Pollen viability was analyzed using five flowers per plant sampled from three different plants per replicate in both years. In the laboratory, pollen grains were spread on microscope slides. Then, one droplet of DAB solution (3.3′ Diaminobenzidine Sigma-Aldrich, St. Louis, MO, USA) was added to each pollen sample; slides were gently warmed with a gas lighter and mounted with a cover slip [50]. Scoring was made using an Leitz Laborlux 12 microscope (Leica, Wetzlar, DE, Germany).

### 4.4. Total Carotenoids, Lycopene, β-Carotene and Chlorophylls

Samples of freshly harvested, fully ripened tomato fruits and leaves were collected from each plot to determine pigments content by a colorimetric assay on freeze-dried and finely ground samples. The evaluation of total carotenoids, chlorophylls, lycopene and β-carotene was carried out according to the method reported by Wellburn et al. [51] and by Zouari et al. [52], and modified by Rigano et al. [53]. To obtain the lipophilic extract, 0.25 g of sample were extracted with 24 mL of acetone/hexane (40/60, *v*/*v*). The mixture was centrifuged at 15,000 rpm for 5 min at 4 °C. Supernatants were collected and stored at −20 °C until analyses. To determine the level of carotenoids and chlorophylls a and b, absorbance of lipophilic extracts was read at 470, 663, and 645 nm, respectively. For lycopene and β-carotene levels’ absorbance was read at 505 and 453 nm, respectively. Three separated biological replicates for each sample and three technical assays for each biological repetition were measured.

### 4.5. Ascorbic Acid Content

Measurements of reduced ascorbic acid (AsA) and total ascorbic acid (AsA + dehydroascorbate—DHA) contents were carried out by using a colorimetric method [54], with modifications reported by Rigano et al. [55,56]. Briefly, 500 mg of frozen powder from tomato fruits or leaves were extracted with 600 µL of 6% trichloroacetic acid (TCA). The mixture was incubated for 15 min on ice and centrifuged at 14,000 rpm for 20 min. For reduced AsA evaluation, to 20 µL of supernatant were added 20 µL of 0.4 M phosphate buffer (pH 7.4), 10 µL of double-distilled (dd) H_2_O and 80 µL of color reagent solution. This solution was prepared by mixing solution A (31% (*w*/*v*) H_3_PO_4_, 4.6% (*w*/*v*) TCA and 0.6% (*w*/*v*) FeCl_3_) with solution B (4% (*w*/*v*) 2,2′ -Dipyridyl). For total AsA, to 20 µL of sample, 20 µL of 5 mM dithiotreitol in 0.4 M phosphate buffer (pH 7.4) was added and the mixture was incubated for 20 min at 37 °C. Ten microliters of N-ethyl maleimide (NEM; 0.5% (*w*/*v*) in water) were added and left for 1 min at room temperature. Eighty microliters of color reagent were added as previously described for reduced AsA. Both the final mixtures were incubated at 37 °C for 40 min and measured at 525 nm using a Nano Photometer TM (Implen, Munich, Germany). The concentration was expressed in mg/100 g of fresh weight (FW). Three separated biological replicates for each sample and three technical assays for each biological repetition were measured.

### 4.6. Antioxidant Activity Determination

The antioxidant capacity was analyzed by FRAP assay carried out by using the ferric reducing/antioxidant power method [57] with slight modifications. The FRAP assay was carried out by adding in a vial 2.5 mL of acetate buffer at pH 3.6, 0.25 mL of TPTZ solution (10 mM) in 40 mM HCl, 0.25 mL of FeCl_3_·6H_2_O solution (12 mM), and 150 µL of methanolic extract obtained by adding 5 mL of 60% methanol base solution to 250 mg of frozen powder. The mixture was incubated for 30 min in the dark, and then readings of the colored products (ferrous tripyridyltriazine complex) were taken at 593 nm using a spectrophotometer. Results were expressed as micromoles of Trolox equivalents (TE) per 100 g FW. Three separated biological replicates for each sample, and three technical assays for each biological repetition, were measured.

### 4.7. Statistical Analysis

Data were subjected to analysis of variance using a two-way ANOVA. To separate means within each parameter, Duncan’s test was performed. Differences of *p* < 0.05 were considered significant. ANOVA was performed by using SPSS (Statistical Package for Social Sciences) Package 6, version 23. A heat map, generated by using the http://biit.cs.ut.ee/clustvis (accessed on 1 April 2021) program package with Euclidean distance as the similarity measure and hierarchical clustering with complete linkage heatmap, summarized all the plant responses to both water-deficit and plant-based biostimulants.

## 5. Conclusions

In this study, a novel protein hydrolysate-based biostimulant was effective at enhancing tomato growth and productivity in different experimental fields, and also under limited water availability. This was possible thanks to the enhancement of the water status of the treated plants coupled with higher antioxidant activity, which are part of a common tolerance strategy generally employed by plants to overcome different abiotic stresses. Even if additional research is needed to fully understand its mechanisms of action, these results can be valuable to functionalize the use of this class of biostimulants in real agricultural contexts, which is necessary to increase their efficacy. Finally, this biostimulant increased fruit quality thanks to the accumulation of antioxidant molecules, including carotenoid and lycopene, which is an added value in regard to the increasing interest in the nutraceutical properties of food.

## Figures and Tables

**Table 1:** Pollen viability, average fruit weight, number of fruits per plant, yield per plant and shoot fresh weight (mean ± SD) in the tomato genotype ‘E42’ treated (biostimulant) or not (control) with the protein hydrolysate and grown in Benevento in the year 2020. Asterisks indicate significant differences according to Student’s *t*-test (** = *p* < 0.01; *** = *p* < 0.001).

	Control	Biostimulant	Significance
Pollen viability (%)	48 ± 0.30	53 ± 0.15	***
Average fruit weight (g)	9.69 ± 0.0017	9.08 ± 0.0003	***
Number of fruits *per* plant	61.87 ± 18.29	139.53 ± 25.77	***
Yield (kg pt^−1^)	0.60 ± 0.18	1.27 ± 0.23	***
Shoot FW (g)	578.33 ± 160.68	966.67 ± 208.77	**

## Data Availability

The data presented in this study are available on request from the corresponding author.

## Supplementary Materials

The following are available online at https://www.mdpi.com/article/10.3390/plants10040783/s1, Table S1: Pollen viability, average fruit weight, number of fruits, fruit water content, yield per plant and shoot fresh weight, (mean ± SD) in E42 tomato plants grown in Battipaglia and treated with the biostimulant under two irrigation regimens. Asterisks indicate significant effect of limited water availability (W), biostimulant treatment (B) and their interaction (W × B) according to ANOVA (ns = not significant; * = *p* < 0.05; ** = *p* < 0.01; *** = *p* < 0.001). Different letters indicate significant differences based on Duncan’s test (*p* ≤ 0.05)., Table S2: Content of total AsA, reduced AsA, carotenoids, chlorophyll a and b (Chl a, b), β-carotene, lycopene and total antioxidant activity (FRAP) (mean ± SD) in leaves and fruit of E42 tomato plants grown in Battipaglia and treated with the biostimulant under two irrigation regimens. Asterisks indicate significant effect of limited water availability (W), biostimulant treatment (B) and their interaction (W × B) according to ANOVA (ns = not significant; * = *p* < 0.05; ** = *p* < 0.01; *** = *p* < 0.001). Different letters indicate significant differences based on Duncan’s test (*p* ≤ 0.05)., Table S3: Cycoflow composition expressed in g/100 g, modified from Francesca et al. (2020)., Figure S1: Daily trends of temperatures (minimum, maximal and average temperature data) during tomato cropping cycle in 2019 and in 2020 in the two different cultivation areas.
