# Supplementary material for: A Novel Protein Hydrolysate-Based Biostimulant Improves Tomato Performances under Drought Stress
Source: Plants (Basel). 2021 Apr 16;10(4):783. doi: 10.3390/plants10040783 (PMC8073256; doi:10.3390/plants10040783)

Supplementary material

Table S1. Pollen viability, average fruit weight, number of fruits, fruit water content, yield *per* plant and shoot fresh weight, (mean± SD) in E42 tomato plants grown in Battipaglia and treated with the biostimulant under two irrigation regimens. Asterisks indicate significant effect of limited water availability (W), biostimulant treatment (B) and their interaction (W x B) according to ANOVA (ns = not significant; * = p < 0.05; ** = p < 0.01; *** = p < 0.001). Different letters indicate significant differences based on Duncan's test (p ≤ 0.05).

	100%		50%		Significance		
	**CONTROL**	**BIOSTIMULANT**	**CONTROL**	**BIOSTIMULANT**	**W**	**B**	**WxB**
Pollen viability (%)	73 ± 12 b	77 ± 10 b	53 ± 8 a	80 ± 8 b	***	**	***
Average fruit weight (g)	7.69 ± 0.73 b	7.57 ± 0.27 b	4.71 ± 1.13 a	9.19 ± 1.24 b	ns	***	**
Number of fruits	162.75 ± 35.13 c	135.25 ± 15.44 c	14.00 ± 3.16 a	47.00 ± 16.10 b	***	ns	**
Fruit water content (%)	98.68 ± 0.72 bc	99.12 ± 0.33 c	91.13 ± 1.15 a	97.84 ± 0.47 b	***	***	***
Yield (kg pt^{-1})	1.25 ± 0.27 c	1.76 ± 0.60 c	0.07 ± 0.02 a	0.44 ± 0.19 b	***	ns	*
Shoot FW (kg)	2.55 ± 0.79	4.23 ±0.05	0.50 ± 0.11	2 ± 0.48	***	***	ns

Table S2. Content of total AsA, reduced AsA, carotenoids, chlorophyll a and b (Chl a, b), β-carotene, lycopene and total antioxidant activity (FRAP) (mean± SD) in leaves and fruit of E42 tomato plants grown in Battipaglia and treated with the biostimulant under two irrigation regimens. Asterisks indicate significant effect of limited water availability (W), biostimulant treatment (B) and their interaction (W x B) according to ANOVA (ns = not significant; * = p < 0.05; ** = p < 0.01; *** = p < 0.001). Different letters indicate significant differences based on Duncan's test (p ≤ 0.05).

	100%		50%		Significance		
**LEAF**	**CONTROL**	**BIOSTIMULANT**	**CONTROL**	**BIOSTIMULANT**	**W**	**B**	**WxB**
Total Asa (mg/100 g FW)	93.51±2.53 b	65.96±9.58 a	101.82±4.80 c	94.35±2.24 b	***	***	***
Reduced AsA (mg/100 g FW)	22.26±0.47 b	15.73±2.47 a	22.81±0.42 b	22.14±2.90 b	***	***	**
Carotenoids (mg/100 g FW)	25.16±3.59	24.11±2.32	26.22±0.33	27.43±0.45	**	ns	ns
Chl a (mg/100 g FW)	132.04±0.92 b	113.09±0.60 a	130.27±3.76 b	129.54±4.45 b	***	***	***
Chl b (mg/100 g FW)	51.02±2.50 b	43.95±4.86 a	51.05±4.67 b	52.67±3.53 b	**	ns	**
FRAP (mmol TE/ 100 g FW)	179.48±18.14 a	202.48±65.77 a	174.38±18.50 a	345.44±66.35 b	**	***	**
**FRUIT**							
Total Asa (mg/100 g FW)	115.40±11.41	100.99±6.68	111.50±7.69	102.70±8.38	ns	**	ns
Reduced AsA (mg/100 g FW)	94.20±4.90 b	84.65±7.15 a	91.11±5.03 ab	94.43±3.37 c	ns	ns	**
Carotenoids (mg/100 g FW)	11.61±0.51 a	15.47±0.95 c	16.58±0.32 d	13.31±0.41 b	***	ns	***
β-Carotene (mg/100 g FW)	0.34±0.05	0.33±0.03	0.40±0.02	0.37±0.07	**	ns	ns
Lycopene (mg/100 g FW)	0.67±0.08 a	0.88±0.06 c	0.90±0.10 c	0.76±0.06 b	ns	ns	***
FRAP (mmol TE/ 100 g FW)	413.55±48.20	426.52±58.38	845.10±79.03	882.24±73.71	***	ns	ns

Table S3. Cycoflow composition expressed in g/100 g, modified from Francesca et al. (2020).

**Glycine betaine**	**3.62**
**Total amino acid**	
Aspartic acid (including asparagine)	2.22
Glutamic acid (including glutamine)	5.04
Alanine	1.36
Arginine	1.06
Phenylalanine	0.83
Glycine	1.02
isoleucine	1.06
Histidine	0.4
Leucine	1.48
Lysine	1.68
Proline	0.81
Serine	1.04
Tyrosine	0.76
threonine	0.98
Valine	1.23
Total cysteine and cystine	0.21
Total tryptophan	0.27
Methionine	0.32
**TOTAL**	**21.77**
**Free Amino Acid**	
Lysine	0.62
Aspartic acid	0.55
Glutamic acid	0.91
Alanine	0.79
Arginine	0.49
Phenylalanine	0.56
Glycine	0.24
isoleucine	0.58
Histidine	0.13
Leucine	0.95
Methionine	0.22
Proline	0.26
Serine	0.43
Tyrosine	0.37
threonine	0.43
Valine	0.72
**TOTAL**	**8.25**
**Micronutrients**	
Boron	0.2
Manganese	1.0
Zinc	1.2

Figure S1. Daily trends of temperatures (minimum, maximal and average temperature data) during tomato cropping cycle in 2019 and in 2020 in two different cultivation areas (Battipaglia and Benevento).

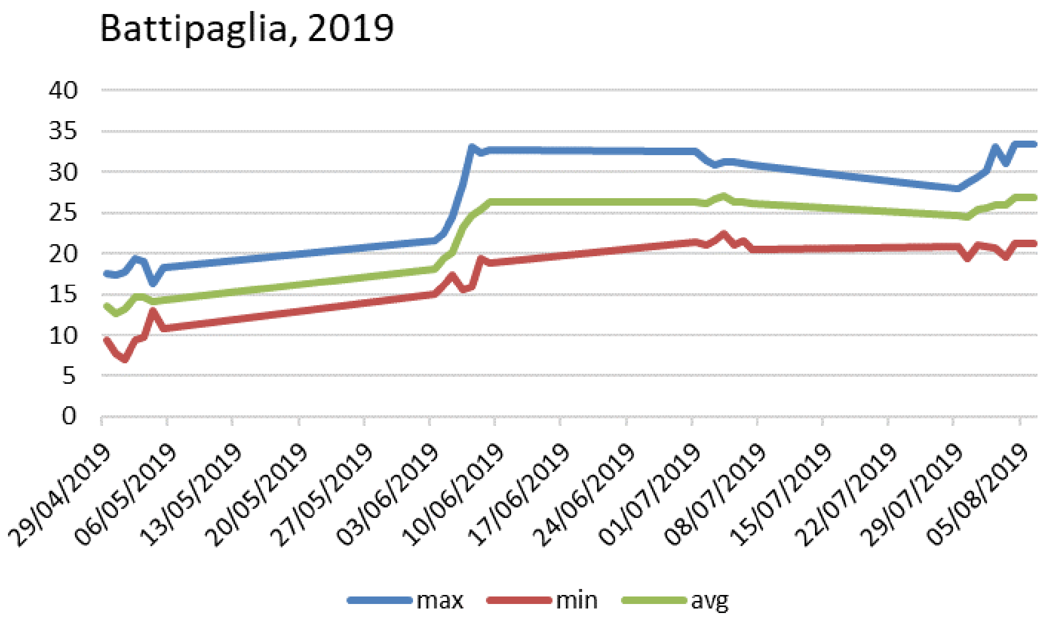

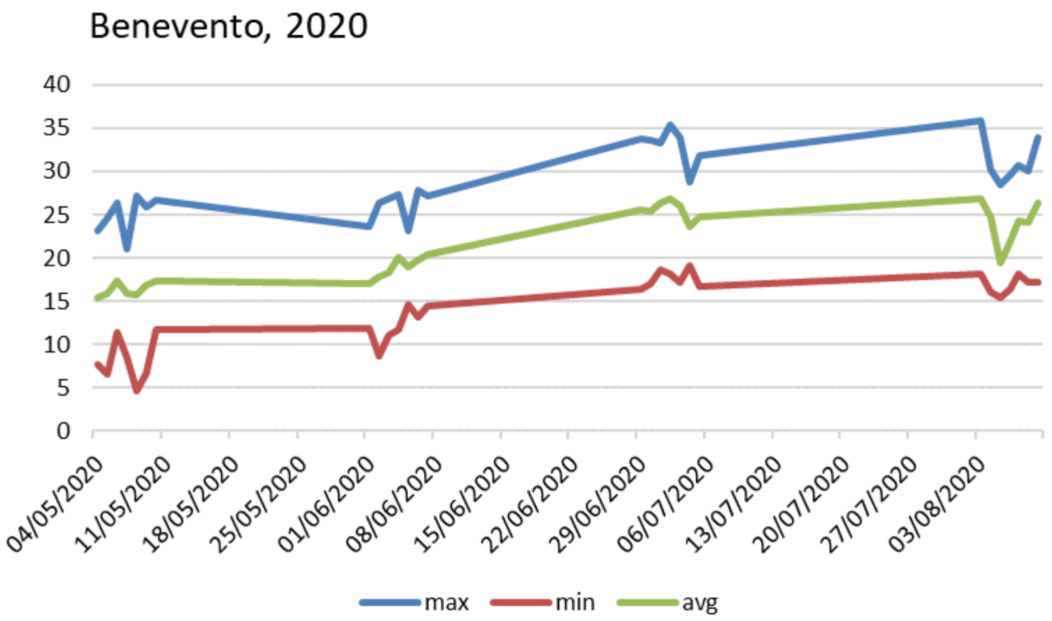

Supplement: Supplementary file 1 [file plants-10-00783-s001.zip › plants-1188810-supplementary.pdf]
